# Biogeography of *Persephonella* in deep-sea hydrothermal vents of the Western Pacific

**DOI:** 10.3389/fmicb.2013.00107

**Published:** 2013-04-25

**Authors:** Sayaka Mino, Hiroko Makita, Tomohiro Toki, Junichi Miyazaki, Shingo Kato, Hiromi Watanabe, Hiroyuki Imachi, Tomo-o Watsuji, Takuro Nunoura, Shigeaki Kojima, Tomoo Sawabe, Ken Takai, Satoshi Nakagawa

**Affiliations:** ^1^Laboratory of Microbiology, Faculty of Fisheries Sciences, Hokkaido UniversityHakodate, Japan; ^2^Subsurface Geobiology Advanced Research Project, Institute of Biogeosciences, Japan Agency for Marine-Earth Science and TechnologyYokosuka, Japan; ^3^Department of Chemistry, Biology, and Marine Science, Faculty of Science, University of the RyukyusNishihara, Okinawa, Japan; ^4^Japan Collection of Microorganisms, RIKEN BioResource CenterTsukuba, Japan; ^5^Marine Biodiversity Research Program, Japan Agency for Marine-Earth Science and TechnologyYokosuka, Japan; ^6^Department of Marine Ecosystem Dynamics, Atmosphere and Ocean Research Institute, The University of TokyoKashiwa, Japan

**Keywords:** population structure, biogeography, deep-sea hydrothermal vent, *Persephonella*, *Aquificales*, MLSA, MALDI-TOF/MS, chemolithoautotroph

## Abstract

Deep-sea hydrothermal vent fields are areas on the seafloor with high biological productivity fueled by microbial chemosynthesis. Members of the *Aquificales* genus *Persephonella* are obligately chemosynthetic bacteria, and appear to be key players in carbon, sulfur, and nitrogen cycles in high temperature habitats at deep-sea vents. Although this group of bacteria has cosmopolitan distribution in deep-sea hydrothermal ecosystem around the world, little is known about their population structure such as intraspecific genomic diversity, distribution pattern, and phenotypic diversity. We developed the multi-locus sequence analysis (MLSA) scheme for their genomic characterization. Sequence variation was determined in five housekeeping genes and one functional gene of 36 *Persephonella hydrogeniphila* strains originated from the Okinawa Trough and the South Mariana Trough (SNT). Although the strains share >98.7% similarities in 16S rRNA gene sequences, MLSA revealed 35 different sequence types (ST), indicating their extensive genomic diversity. A phylogenetic tree inferred from all concatenated gene sequences revealed the clustering of isolates according to the geographic origin. In addition, the phenotypic clustering pattern inferred from whole-cell matrix-assisted laser desorption ionization-time of flight mass spectrometry (MALDI-TOF/MS) analysis can be correlated to their MLSA clustering pattern. This study represents the first MLSA combined with phenotypic analysis indicative of allopatric speciation of deep-sea hydrothermal vent bacteria.

## Introduction

Mixing of hydrothermal fluids and ambient seawater at the seafloor creates physically and chemically dynamic habitats for microorganisms. Vent fluids physicochemistry is variable both spatially and temporally as a result of subsurface geological and geochemical processes (Edmond et al., 1979; Butterfield and Massoth, 1994; Butterfield et al., 2004). Diverse microorganisms including both Archaea and Bacteria have been isolated in pure cultures from various hydrothermal fields (Nakagawa and Takai, 2006). In addition, culture-independent studies revealed the dominance of yet-to-be cultured microorganisms in deep-sea hydrothermal environments (Haddad et al., 1995; Takai and Horikoshi, 1999; Reysenbach et al., 2000; Corre, 2001; Teske et al., 2002), and provided insight into the great heterogeneity of microbial communities between hydrothermal systems. The heterogeneity can be correlated to differences in the geological and chemical properties between different vents (Takai et al., 2004; Nakagawa et al., 2005a,b; Takai and Nakamura, 2011). On the other hand, there are also some cosmopolitan genera found in deep-sea hydrothermal systems occurring not only in the Mid-Ocean Ridge systems but in the Back-Arc Basin systems and the Volcanic Arc systems (Takai et al., 2006; Nakagawa and Takai, 2008; Kaye et al., 2011). Members of the genus *Persephonella* belonging to the order *Aquificales*, obligately sulfur- and/or hydrogen-oxidizing, chemoautotrophic, thermophilic bacteria, are widely distributed in deep-sea hydrothermal systems (Reysenbach et al., 2000, 2002; Takai et al., 2004; Nakagawa et al., 2005a,b; Ferrera et al., 2007; Takai et al., 2008). Although the widespread occurrence of this group suggests that they may play important role, many questions remained about their physiology, metabolism, and ecology within the environment because of the difficulty in isolating these strains. Some isolates have been characterized (Götz et al., 2002; Nakagawa et al., 2003), and implied their role in carbon, sulfur and nitrogen cycles in high temperature habitats at deep-sea vents (Reysenbach et al., 2002; Ferrera et al., 2007). However, little is known about the spatial or biogeographical pattern of *Persephonella* microdiversity and phenotypic heterogeneity.

Weak biogeographical signals in microbial communities are usually explained by the hypothesis of microbial cosmopolitanism formulated by Bass Becking (Wit and Bouvier, 2006). However, recent studies have explored the effects of dispersal limitation on microbial biogeography. Like macroorganisms, the genetic similarity negatively correlated with geographic distance, i.e., distance-decay relationship, have been reported for cyanobacteria, sulfate-reducing bacteria, marine planktonic bacteria, and hyperthermophilic archaea (Papke et al., 2003; Whitaker et al., 2003; Vergin et al., 2007; Oakley et al., 2010). In addition, the biogeographical diversity pattern was reported in detail for members of the “deep-sea hydrothermal vent euryarchaeota 2” (Flores et al., 2012). Microbial biogeographical studies have been usually based solely on genetic data. Microbial biogeography was recently studied at the phenotypic level (Rosselló-Mora et al., 2008), however, genetic and phenotypic correlation has not been explored. We investigated the spatial diversity pattern of *Persephonella* population by the combined use of comparative genetic and phenotypic characterizations.

## Materials and methods

### Field site and sampling

**Figure 1:**
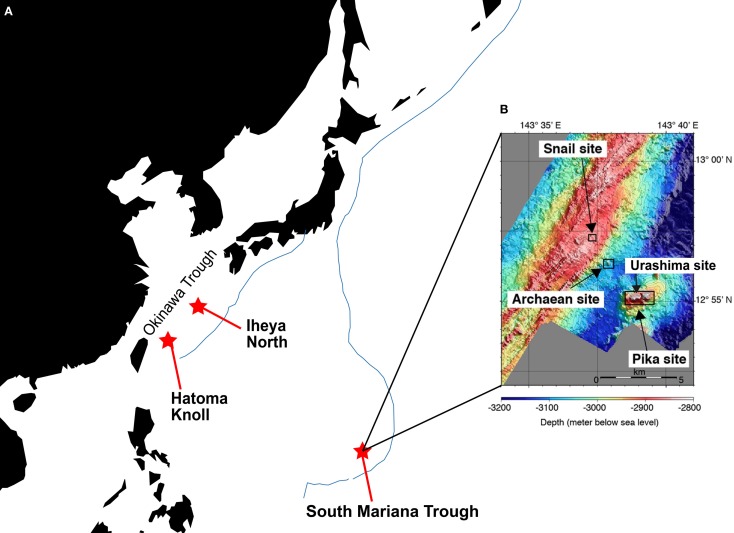
**Sampling sites. (A)** Location of the Iheya North field and Hatoma Knoll in the Okinawa Trough, and the South Mariana Trough. Blue line indicates subduction zone. **(B)** Location of four vent sites in the South Mariana Trough.

Samples, i.e., chimney structures, fluids, and sediments, were collected with R/V Natsushima and ROV Hyper-Dolphin or R/V Yokosuka and DSV Shinkai 6500 from the Okinawa Trough (OT) in 2007 and 2009, or the South Mariana Trough (SMT) in 2010 (Table 1). Vent fluids from the OT are characteristic in the high contents of methane and carbon dioxide (Kawagucci et al., 2011). Among the OT hydrothermal fields, this study focused on the Iheya North and Hatoma Knoll (Figure 1). In the SMT, four vent sites were studied (Figure 1). The Archaean site is located at a ridge flank, about 2 km apart from the backarc-spreading axis. Discharging fluids (*T*_max_ = 318°C) was acidic and depleted in Cl^−^ (Cl^−^ = 401 mM) (Ishibashi et al., 2006). Pika site is located on an off-axis knoll, about 5 km from the axis. Fluid chemistry (*T*_max_ = 330°C) of Pika site showed brine-rich signature (Cl^−^ = 600 mM) (Ishibashi et al., 2006). Urashima site is newly discovered in 2010, and located at the northern foot of the western peak of the same knoll as Pika. Snail site is located on the active backarc-spreading axis. After retrieval on board, each of the chimney structures were sectioned immediately into the exterior surface and the inside parts, and slurried with 25 ml of sterilized seawater in the presence or absence of 0.05% (w/v) neutralized sodium sulfide in 100 ml glass bottles (Schott Glaswerke, Mainz, Germany). Bottles were then tightly sealed with butyl rubber caps under a gas phase of 100% N_2_ (0.2 MPa). Similarly, fluid, sediment, and biological samples were prepared anaerobically in 10 ml glass bottles. Samples were stored at 4°C until use.

**Table 1:** **Information of samples and cultivation temperature**.

**Strains**	**Originated samples**	**Origins**	**Sodium sulfide**	**Isolated temperature (°C)**	**Sequence similarity to *P. hydrogeniphila* 29W^T^ (%)**
OT-1	CS	Iheya North, OT	+	55	99.7
OT-2	CS	Iheya North, OT	−	55	99.6
OT-3	CI	Iheya North, OT	+	55	98.9
OT-4	CS	Iheya North, OT	−	55	99.6
OT-5	CS	Hatoma Knoll, OT	−	55	99.7
MT-6	CI	Urashima, SMT	−	55	99.6
MT-7	CI	Urashima, SMT	+	55	99.5
MT-8	CS	Urashima, SMT	−	55	99.5
MT-9	CS	Urashima, SMT	+	55	99.5
MT-10	CI	Urashima, SMT	+	55	99.7
MT-11	CA	Urashima, SMT	+	55	99.5
MT-12	CS	Urashima, SMT	+	55	99.5
MT-13	CI	Urashima, SMT	−	47	99.6
MT-14	CS	Urashima, SMT	+	47	99.5
MT-15	CS	Urashima, SMT	+	70	99.5
MT-16	CI	Urashima, SMT	−	70	99.6
MT-17	CS	Urashima, SMT	−	55	99.6
MT-18	CS	Urashima, SMT	+	55	99.5
MT-19	CA	Urashima, SMT	+	55	98.7
MT-20	CA	Urashima, SMT	+	47	98.7
MT-21	CA	Urashima, SMT	+	70	99.5
MT-22	SE	Snail, SMT	−	55	99.5
MT-23	HR	Snail, SMT	−	55	99.5
MT-24	HR	Snail, SMT	+	55	99.6
MT-25	CS	Archaean, SMT	−	55	98.7
MT-26	CI	Archaean, SMT	+	55	98.7
MT-27	CA	Archaean, SMT	−	55	99.5
MT-28	CS	Archaean, SMT	−	55	99.7
MT-29	BS	Archaean, SMT	−	55	99.5
MT-30	FW	Archaean, SMT	−	55	99.5
MT-31	CS	Archaean, SMT	−	55	99.5
MT-32	CS	Archaean, SMT	+	55	99.6
MT-33	CA	Archaean, SMT	−	55	99.7
MT-34	CS	Archaean, SMT	−	47	99.5
MT-35	CS	Pika,SMT	−	55	99.5
MT-36	CI	Pika,SMT	+	55	99.5

*CS, chimney surface; CI, chimney inside parts; CA, all of chimney; SE, sediment sample; HR, rock influenced by vent fluids; FS, fluid sample; BS, biological sample; +, presence of sodium sulfide; −, absence of sodium sulfide. Sequence similarity was based on 16S rRNA gene sequences of each strain*.

### Enrichment, isolation, and phylogenetic analysis

Serial dilution cultures were performed using the MMJHS medium (Takai et al., 2003) containing a mixture of electron donors and electron acceptors for hydrogen/sulfur-oxidizing chemoautotrophs at 47, 55 and 70°C. MMJHS medium included 1 g each of NaHCO_3_, Na_2_S_2_O_3_.5H_2_O, and NaNO_3_, 10 g of S^0^ and 10 ml vitamin solution (Balch et al., 1979) per liter of MJ synthetic seawater [gas phase: 80% H_2_ +20% CO_2_ (0.3 MPa)]. To obtain pure cultures, dilution-to-extinction was repeated at least 2 times (Baross, 1995). The purity was confirmed routinely by microscopic examination and by sequencing of the 16S rRNA gene using several PCR primers. Genomic DNA was extracted from isolates using the UltraClean Microbial DNA isolation Kit (MoBio Laboratories, Inc., Solana Beach, CA, USA) following the manufacturer's protocol. The 16S rRNA gene of each isolate was amplified by PCR using LA Taq polymerase (TaKaRa Bio, Otsu, Japan) as described previously (Takai et al., 2001). The primers used were Eubac 27F and 1492R (Weisburg et al., 1991). These amplicons were bidirectionally determined by the dideoxynucleotide chain-termination method. Almost complete sequences of the 16S rRNA gene were assembled using Sequencher ver 4.8 (Gene Codes Corporation, Ann Arbor, MI, USA). In order to determine the phylogenetic positions of isolates, the sequences were aligned using Greengenes NAST alignment tool (DeSantis et al., 2006), and compiled using ARB software version 03.08.22 (Ludwig et al., 2004).

### Multi-locus sequence analysis

Intraspecies diversity among isolates was evaluated using multi-locus sequence analysis [MLSA; formerly called multilocus sequence typing (MLST)] technique (Gevers et al., 2005). MLSA represents a universal and unambiguous method for strain genotyping, population genetics, and molecular evolutionary studies (Whitaker et al., 2005; Mazard et al., 2012). Genes selected for MLSA were *tkt* (transketolase), *atpA* (ATP synthase, A subunit), *dnaK* (Hsp 70 chaperon protein), *napA* (nitrate reductase, large subunit), *metG* (methionyl-tRNA synthetase), and *gyrB* (DNA gyrase, B subunit). Primers (Table 2) were designed according to the published complete genome sequences of *Aquificales* members, i.e., *Persephonella marina* EX-H1^T^ (NC_012439) (Reysenbach et al., 2009), *Sulfurihydrogenibium azorense* (NC_012438) (Reysenbach et al., 2009), *Sulfurihydrogenibium* sp. YO3AOP1 (NC_010730) (Reysenbach et al., 2009), *Hydrogenobaculum* sp. Y04AAS1 (NC_011126) (Reysenbach et al., 2009), *Aquifex aeolicus* VF5 (NC_000918) (Deckert et al., 1998), and *Hydrogenivirga* sp. 128-5-R1-1 (NZ_ABHJ01000000) (Reysenbach et al., 2009). ClustalX version 2.0 was used for the alignment of nucleotide sequences (Larkin et al., 2007). PCR were performed under the following conditions: 96°C for 1 min, 35–38 cycles of 96°C for 20 s, annealing for 45 s at temperatures shown in Table 2, and 72°C for 2 min. The PCR products were confirmed by 1% agarose gel electrophoresis and purified with exonuclease I and shrimp alkaline phosphatase. If necessary, bands were excised and purified using Wizard® SV Gel and PCR Clean-up System (Promega, Madison, WI, USA). Purified PCR products were used as templates for Sanger sequencing reaction. The sequences were assembled and edited using Sequencher ver 4.8, and aligned with ClustalX. The sequences were translated into amino acid using Transeq program (EMBOSS; European Molecular Biology Open Software Suite).

**Table 2:** **Primers and PCR conditions for MLSA**.

**Genes**	**Primer name**	**Primers (5′−3′)**	**Primer combination and annealing temperature (°C)**	**Amplicon size (bp)**
*atpA*	atpA101F	ADGTDGGWGAYGGTGTYGC	101F-1081R: 55	980
	atpA1081R	CGTTRATAGCWGGTCTDATACC		
	atpA983R	ACGTCACCBGCYTKWGTTTC	101F-983R: 52	882
*dnaK*	dnaK464F	GDCARGCWACMAARGAYGC	55	741
	dnaK1118R	GCMACWACYTCRTCDGG		
*gyrB*	gyrB377F	CTYCAYGGWGTWGGWGC	52	1186
	gyrB1563R	TCWACRTCRGCRTCHGYCAT		
*napA*	napA181F	TTCTGTGGWACDGGWTGYGG	181F-1713R: 59	1532
	napA690F^*^	ATGGCWGARATGCAYCC		
	napA1714R	CKBGCHGCTTTRATCCARTG		
	napA1347R^*^	GRTTVAWWCCATTGTCCA		
*metG*	metG145F	ACRGGWACMGATGARCATGG	55	686
	metG831R	GCDGGCCARTAWACDGYATG		
*tkt*	tkt478F	AWSGSYGTRGGTATGGC	52	1063
	tkt1541R	TGKGTWGGDCCRTCYTC		

* *Primers used for sequencing reaction*.

*Z*-test, non-synonymous (Ka)/synonymous (Ka) substitution, and Tajima's *D* test were performed as described elsewhere (Vergin et al., 2007). Briefly, values of Ka and Ks were determined using the software program SWAAP ver 1.0.3 (Pride, 2000), set to the Li method with a window size of 90 and step size of 18. *Z*-test was performed using MEGA ver 5.05 software (Tamura et al., 2011) with the following options: purifying selection, overall average, 1000 bootstrap replicates, pairwise deletion and the Pamilo–Bianchi–Li method. Tajima's D based on the total number of mutation were calculated using DnaSP ver 5 (Librado and Rozas, 2009). The combination of allele types for each isolate defined the sequence type (ST). Phylogenetic trees were constructed by the maximum likelihood (ML) method using MEGA ver 5.05. ML bootstrap support was computed after 100 reiterations. Split decomposition trees were constructed with SplitsTree ver 4 using the Neighbor-Net algorithm (Huson, 1998).

The levels of genetic variation within and between populations were calculated with the Arlequin ver 3.5 software (Excoffier and Lischer, 2010). *F*_ST_ values were estimated for groups of two or more strains and were tested for significance against 1000 randomized bootstrap resamplings. Average pairwise genetic distance and standard error based on 500 bootstrap resamplings of each population were estimated using MEGA ver 5.05. Mantel test was performed with XLSTAT software (www.xlstat.com). Sequences obtained in this study have been deposited in DDBJ/EMBL/GenBank under Accession No. AB773894-AB774147.

### Geochemical analysis

Chemical compositions listed in Table 6 were analyzed as previously described (Takai et al., 2008; Toki et al., 2008). End-member fluids compositions were estimated by the conventional method, that is extrapolation to *Mg* = 0 of linear relationship of concentration of each species to Mg among the obtained samples (Von Damm et al., 1985).

### Preparation of bacterial samples for whole-cell MALDI-TOF/MS

Samples for whole-cell matrix-assisted laser desorption ionization-time of flight mass spectrometry (MALDI-TOF/MS) were prepared as described in Hazen et al. (2009). Briefly, *Persephonella* strains were cultured in 3 ml of MMJHS medium at their isolated temperatures (Table 1). Following incubation, cells were washed once in 1 ml of 0.85% NaCl and twice in 1 ml of 50% ethanol at 4°C. Cell pellets were weighed and resuspended in 1% trifluoroacetic acid (TFA) to yield a final concentration of 0.2 mg cells/μl of 1% TFA. Equal volumes of the TFA bacterial suspension and the MALDI-TOF/MS matrix solution (10 mg/ml sinapinic acid in 50% acetonitrile, 50% water, and 0.1% TFA) were mixed in a microcentrifuge tube, and then 1.0 μl of this mixture was spotted in triplicate on a stainless steel MALDI-TOF/MS sample plate (corresponding to approximately 1.4 × 10^8^ cells/spot). Samples were allowed to air dry before being loaded in the mass spectrometer.

### MALDI-TOF/MS and data processing

All mass spectra were acquired using the MALDI-TOF/MS spectrometer (4700 proteomics analyzer; Applied Biosystems, Foster City, CA, USA) in the linear and positive-ion modes. The laser (N_2_, 337 nm) intensity was set above the ion generation threshold. Mass spectra were recorded in the m/z range of 2000–14,000. The acceptance criteria, based on 1000 laser shots per spot, were signal intensities between 2000 and 55,000 counts and a signal/noise ratio of 10 or greater.

Raw mass spectra from three spots were normalized using Data Explorer software (Applied Biosystems, Foster City, CA, USA) by baseline correction and combined to generate an averaged peak list. The peaks around 2000 m/z were excluded as noise.

The peaks were ranked according to their signal intensities, and the top 15 most intense peaks were chosen for further analysis. The relative intensity ratio was calculated for the 15 peaks. Squared distance was estimated based on the presence or absence of peaks by Ward's minimum variance method using MVSP software ver 3.21 (Kovach Computing Services, Wales, UK). The presence or absence of peaks was determined within a tolerance of 14 Da.

## Results

### Isolation of *Persephonella* strains

**Figure 2:**
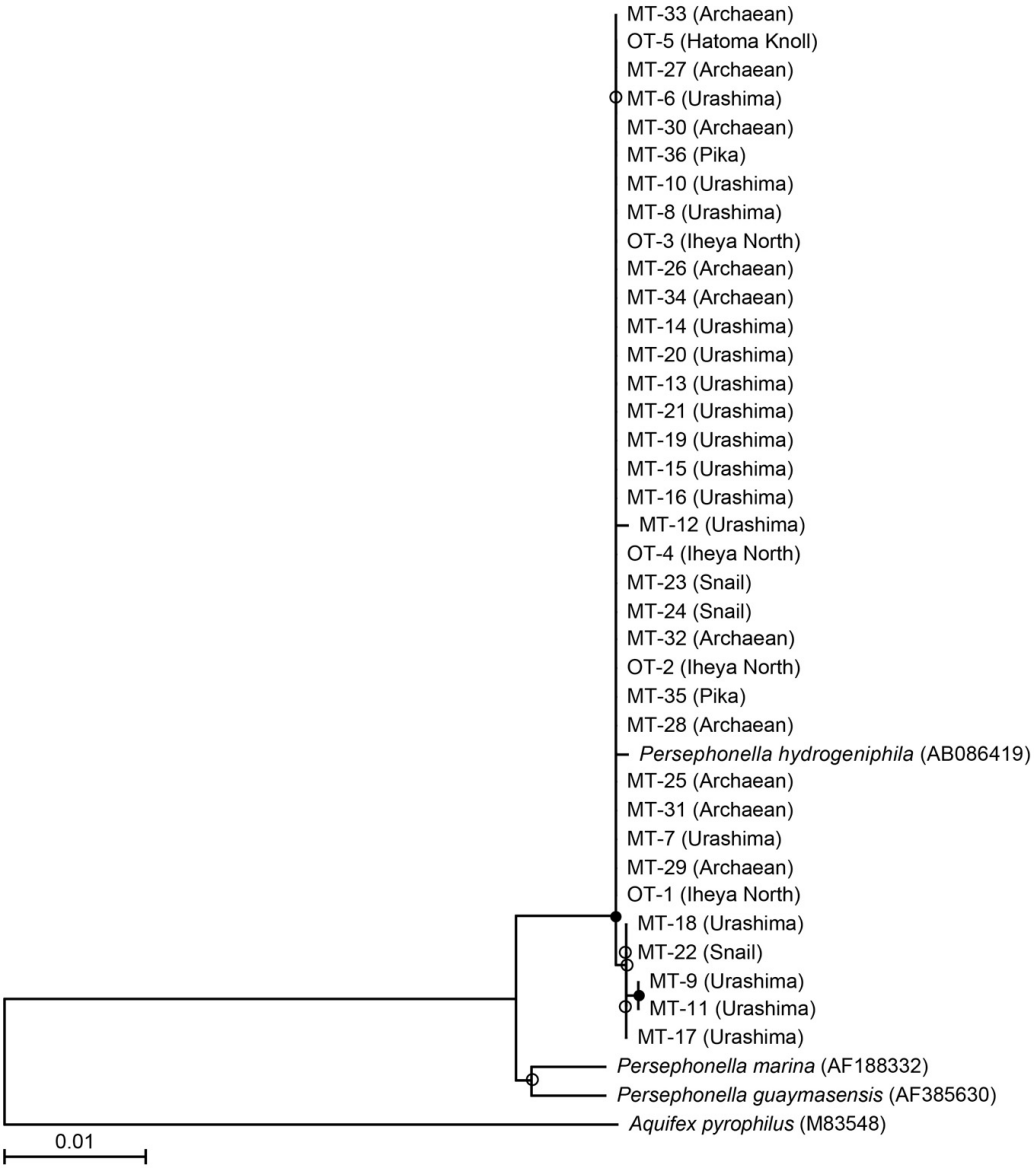
**Phylogenetic relationship of isolates and representative *Persephonella* species as determined by neighbor-joining analysis of 16S rRNA gene sequences.** Tree was constructed by using 1119 sites that could be unambiguously aligned. Origins of isolates and DDBJ accession numbers are shown in parentheses. Branch points conserved with bootstrap values of >75% (filled circle) and bootstrap value of 50–74% (open circles) are indicated.

We investigated a total of 36 *Persephonella* strains originating from various hydrothermal samples from the OT (4 strains from Iheya North, and 1 strain from Hatoma Knoll) and the SMT (16 strains from Urashima site, 10 strains from Archaean site, 3 strains from Snail site, and 2 strains from Pika site) (Figure 2 and Table 1). All of the 36 strains shared >98.7% 16S rRNA gene similarities with one another and with *P. hydrogeniphila* 29W^T^.

### Genetic diversity of *Persephonella* population

We developed a MLSA scheme for the *Persephonella* population based on five housekeeping genes and a functional gene. The gene fragments sequenced varied from 501 to 882 bp in length (Table 1), and nucleotide sequence similarity at MLSA loci varied from 94.6 to 96.3% (average 95.8%). We obtained concatenated sequences of 4254 bp and identified a total of 702 variable positions. Ratios of non-synonymous to synonymous substitutions (*Ka/Ks*) were much smaller than 1 for all loci (Table 3), indicating the genes were subject to purifying selection, conforming to the general requirements for MLSA loci (Maiden, 2006). This was statistically supported by the high values from the *Z*-test (Table 3).

**Table 3:** **Genetic features at the six MLSA loci**.

**Gene**	**Sequence length**	**Nucleocid identity**	**Amino acid identity**	**Ka/Ks**	**Theta**	***Z***	**Tajima's D**
*tkt*	810	95.8 (6.5)	97.5 (4.12)	0.0131 (0.0215)	0.05286	7.4	−0.77603
*atpA*	669	96.3 (5.5)	99.7 (0.45)	0.0062 (0.1535)	0.04456	7.4	−0.59903
*dnaK*	495	94.6 (9.1)	98.0 (8.04)	0.0024 (0.0461)	0.11838	4.8	−2.05655
*napA*	882	94.8 (4.3)	97.9 (2.75)	0.0581 (0.0812)	0.05326	8.9	−0.06984
*metG*	555	95.9 (6.0)	98.4 (2.48)	0.0381 (0.0754)	0.04858	5.3	−0.61694
*gyrB*	843	95.6 (6.1)	99.5 (0.68)	0.0146 (0.0269)	0.05223	7.1	−0.57104

*Average nucleotide identities (ANI) and synonymous (Ks) vs. non-synonymous (Ka) substitutions were determined from 36 different Persephonella isolates*.

### Population genetic structure

Typing based on sequences of six protein-coding gene fragments revealed 35 different STs among 36 isolates, indicating the high genetic diversity of *Persephonella* population (Table 4). The number of different alleles per locus varied between 16 for *metG* and 24 for *tkt*. Strains MT-17 and −18 had identical sequences for all MLSA loci. These strains were isolated from the same chimney sample, but the slurries were prepared in the presence (used for strain MT-18) or absence (used for strain MT-17) of 0.05% (w/v) sodium sulfide. In other cases, the presence of sodium sulfide in slurries resulted in the isolation of strains classified into different STs (Table 1).

**Table 4:** **Allelic properties at six loci of *Persephonella* isolates analyzed in this study**.

**Strains**	**Sequence type**	**Allele no. at locus**						**Origin**
		***atpA***	***dnaK***	***gyrB***	***metG***	***napA***	***tkt***	
MT-17,18	1	1	1	1	1	2	2	Urashima, SMT
MT-31	2	1	7	6	3	3	3	Archaean, SMT
MT-33	3	1	2	2	1	4	3	Archaean, SMT
MT-22	4	1	11	12	11	10	14	Snail, SMT
MT-11	5	2	4	2	4	5	5	Urashima, SMT
MT-28	6	2	5	3	10	4	12	Archaean, SMT
MT-12	7	2	10	11	5	5	13	Urashima, SMT
MT-25	8	2	2	3	6	5	3	Archaean, SMT
MT-10	9	2	2	19	1	15	2	Urashima, SMT
MT-6	10	2	13	15	5	5	16	Urashima, SMT
MT-9	11	2	4	2	4	14	21	Urashima, SMT
MT-35	12	3	4	3	4	3	4	Pika,SMT
MT-27	13	3	5	3	4	3	8	Archaean, SMT
MT-16	14	4	6	4	4	13	6	Urashima, SMT
MT-13	15	4	6	4	4	6	6	Urashima, SMT
MT-15	16	4	14	4	4	6	19	Urashima, SMT
MT-20	17	5	3	16	2	2	17	Urashima, SMT
MT-21	18	5	15	18	2	2	2	Urashima, SMT
MT-7	19	6	2	3	1	1	4	Urashima, SMT
MT-8	20	6	2	3	1	1	20	Urashima, SMT
MT-29	21	7	2	5	7	1	1	Archaean, SMT
MT-19	22	8	3	1	2	2	2	Urashima, SMT
MT-32	23	9	8	7	8	4	4	Archaean, SMT
MT-23	25	10	4	8	1	8	9	Snail, SMT
MT-24	26	11	2	9	9	9	10	Snail, SMT
MT-36	27	12	9	10	1	3	11	Pika, SMT
MT-30	28	13	12	3	1	11	5	Archaean, SMT
MT-26	30	14	2	13	6	12	1	Archaean, SMT
MT-14	31	15	4	3	3	3	3	Urashima, SMT
MT-34	32	16	4	17	1	3	18	Archaean, SMT
OT-1	33	17	16	20	12	7	22	Iheya North, OT
OT-2	34	18	17	21	13	16	23	Iheya North, OT
OT-4	35	19	18	22	14	7	24	Iheya North, OT
OT-3	36	20	19	23	15	17	7	Iheya North, OT
OT-5	37	21	20	14	16	18	15	Hatoma Knoll, OT

**Figure 3:**
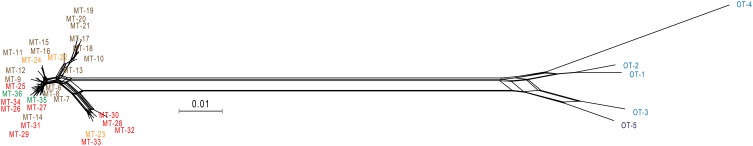
**Neighbor-Net graph based on the concatenated sequences of 6 protein-coding genes of *Persephonella* isolates showing a bushy network structure indicative of homologous recombination.** Scale bar represent 0.1 substitutions per nucleotide position. Origins of isolates are indicated as follows: light blue, Iheya North; blue, Hatoma Knoll; red, Archaean; orange, Snail; green, Pika; brown, Urashima.

The split graph obtained from the concatenated sequence data displayed bushy network structures with complex parallelogram formation indicative of extensive homologous recombination (Figure 3). The result of PHI test (Bruen et al., 2006) for the concatenated sequences also showed the presence of the past recombination events during the evolution of *Persephonella* (*p* < 0.05).

### Population difference between the OT and the SMT

**Figure 4:**
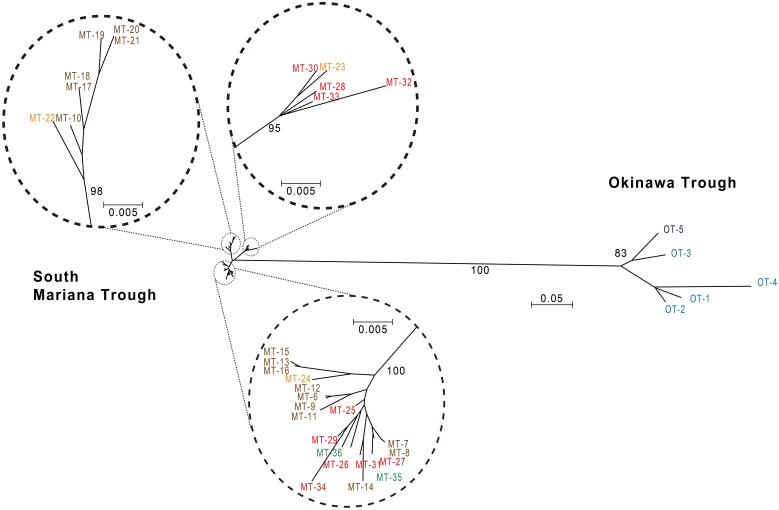
**Maximum likelihood tree based on concatenated sequences of all MLSA loci.** Origins of isolates are indicated as follows: light blue, Iheya North; blue, Hatoma Knoll; red, Archaean; orange, Snail; green, Pika; brown, Urashima.

A ML phylogenetic tree derived from the concatenated alignment of six loci showed two different clades with high bootstrap support (Figure 4). The two clades corresponded to the two geographic regions, showing that the SMT strains share a common evolutionary history distinct from the OT strains. The *F*_ST_ value confirmed that the OT and the SMT populations were significantly different (*F*_ST_ = 0.8711, *p* < 0.05) (Table 5).

**Table 5:** ***F*_ST_ values between each population**.

	**Snail**	**Archaean**	**Pika**	**Urashima**	**SMT**	**OT**
Snail	0.0000					
Archaean	−0.0173	0.0000				
Pika	−0.4221	−0.3450	0.0000			
Urashima	−0.0130	**0.1200**	−0.1909	0.0000		
SMT	−	−	−	−	0.0000	
OT	−	−	−	−	**0.8711**	0.0000

*P-values <0.05 are bold*.

### Correlation between chemistry and genetic diversity

**Figure 5:**
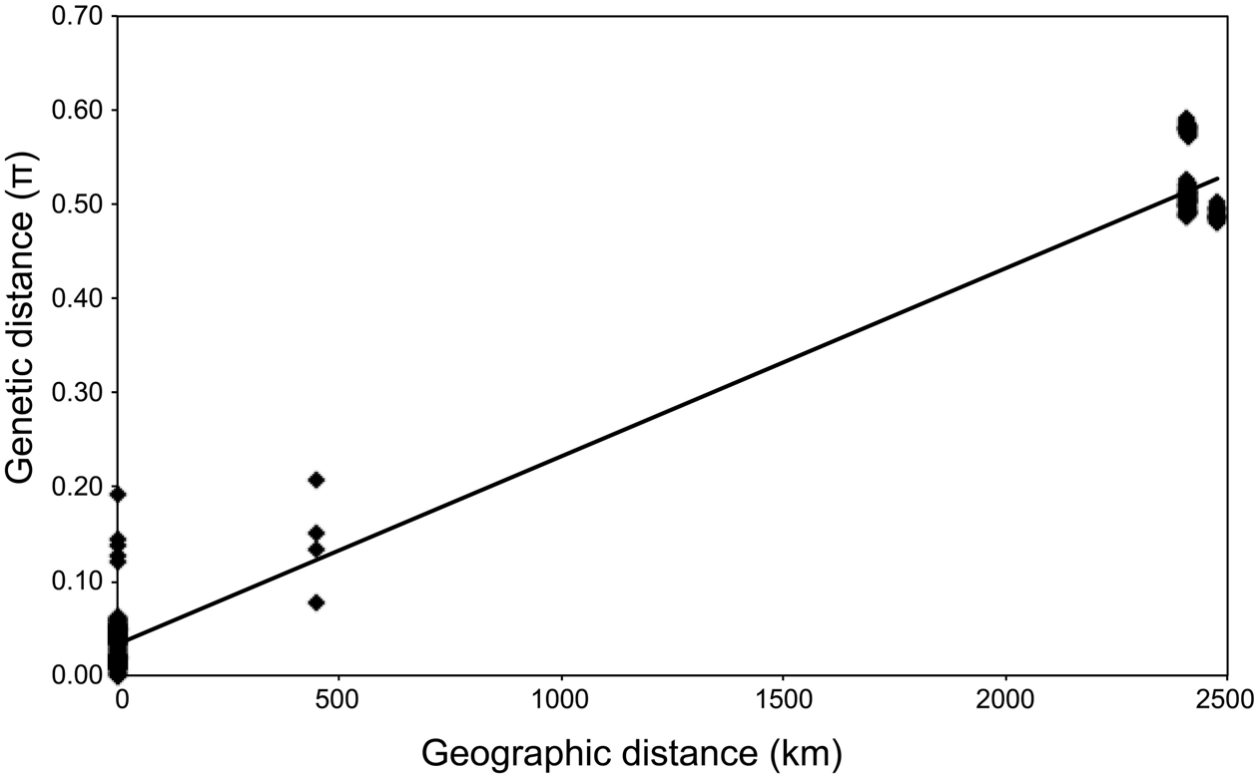
**Relationship between genetic and geographic distance (*R*^2^ = 0.98).** π, number of base substitutions per site from between sequences.

Geochemical analysis revealed that different vent fluids had distinctive end-member chemical compositions (Table 6). Although the vent fluids from Archaean and Pika were respectively Cl^−^-depleted and -enriched in 2004 and 2005 (Ishibashi et al., 2006), no significant difference was found between them in this study. We assessed the relative contributions of environmental factors (such as pH and maximum temperature of vent fluids) and geographic distance to *Persephonella* genetic structure using the Mantel test. The pH of SMT vent fluids (pH 3.0–3.5) were significantly lower than those (pH 5.0–5.2) of OT (Table 6). However, we found no significant correlation between the genetic distance and the absolute difference in vent fluid pH and temperature (Mantel *r* = −0.28, *p* = 0.4). In contrast, a large, significant correlation coefficient (Mantel *r* = 0.993, *p* < 0.0001) was found in a Mantel test of all pairwise comparisons of the genetic and the geographic distance between strains (Figure 5).

**Table 6:** **End-member compositions of vent fluids from the OT and the SMT**.

**Venting sit**	**Sampling**	Tmax^*3^	pH^*4^	**Cl^−^**	**Na**	**K**	**Ca**	**Mn**	**NH^+^_4_**	**SO^2−^_4_**	**H_2_S**
	**year**	**°C**		**mmol/kg**	**mmol/kg**	**mmol/kg**	**mmol/kg**	**μ mol/kg**	**μ mol/kg**	**mmol/kg**	**mmol/kg**
Iheya North^*1^ (NBC)	2007	309	5.0	557	407	72.4	21.9	6.58 × 102	1.71 × 103	0	–
Hatoma Knoll^*2^	2000	240	5.2	381	285	54.6	17.0	4.83 × 102	7.20 × 103	–	–
Urashima	2010	280	3.0	623	456	37.0	31.9	2.22 × 103	<100	−4.11	2.4
Snail	2010	61	3.5	558	442	28.4	28.0	1.84 × 103	<100	−0.41	2.0
Archaean	2010	318	3.0	401	312	33.0	15.8	1.28 × 103	<100	−0.24	9.6
Pika	2010	322	3.0	469	444	31.6	37.8	1.14 × 103	<100	−2.98	7.0

*1 *Kawagucci et al. (2011)*.

*2 *Kishida et al. (2004)*.

*3 *Maximum temperature*.

*4 *Measured at 25°C*.

*−, No data*.

### Whole-cell MALDI-TOF/MS analysis

**Figure 6:**
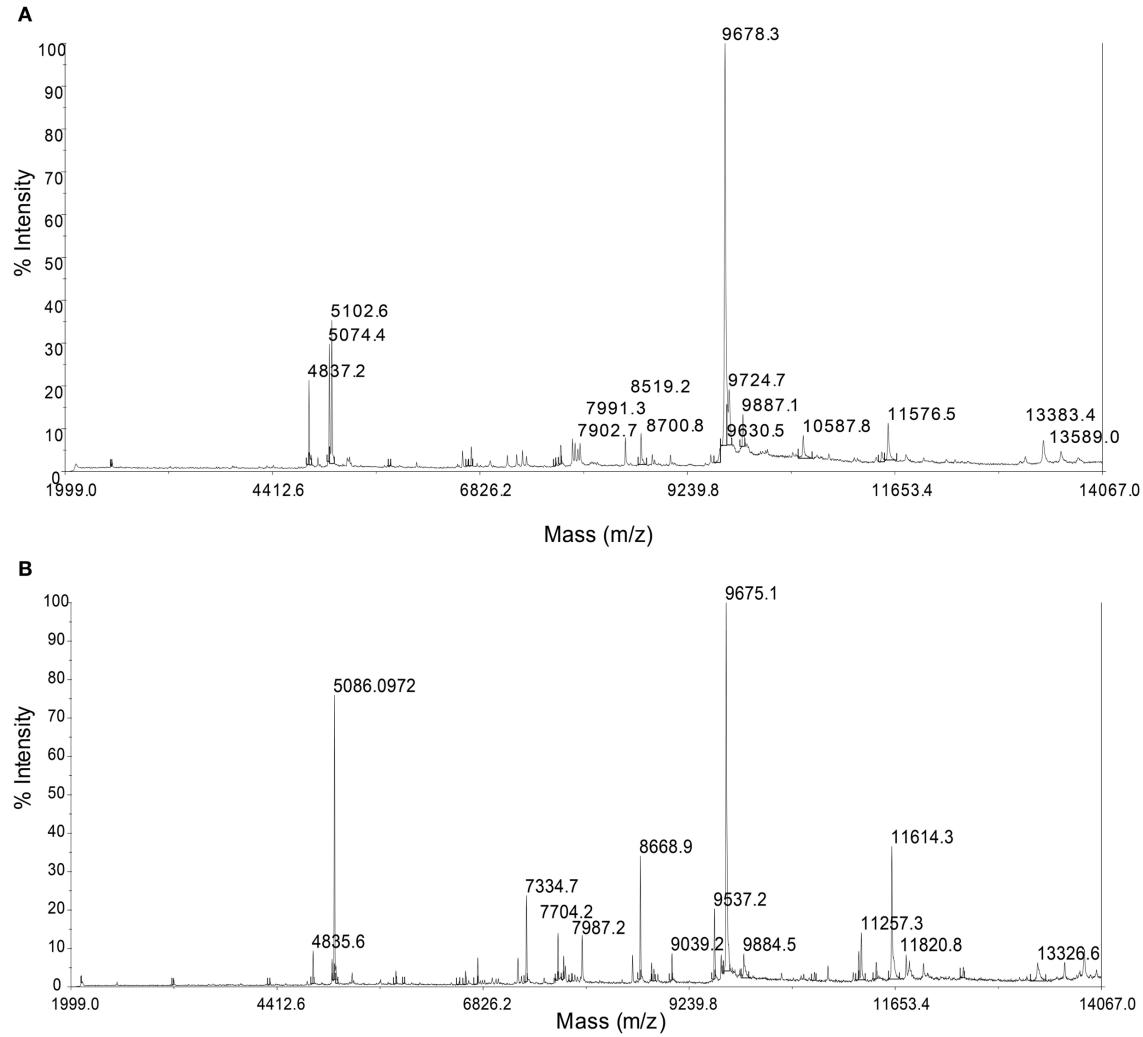
**Representative whole-cell MALDI-TOF/MS spectra from the OT strain (OT-2, A) and the SMT strain (MT-26, B)**.

**Figure 7:**
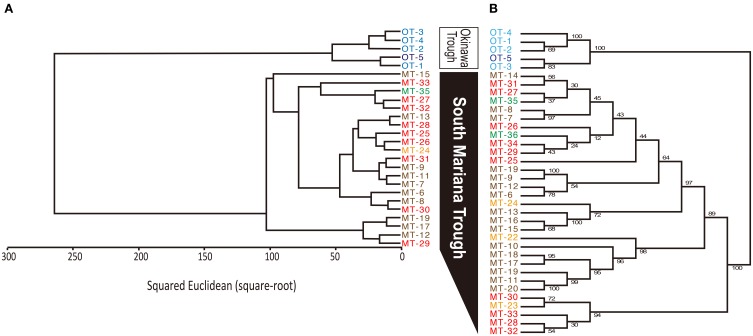
**Correlation between genotype and phenotype inferred from MALDI-TOF/MS analysis (A) and MLSA (B).** Origins of isolates are indicated as follows: light blue, Iheya North; blue, Hatoma Knoll; red, Archaean; orange, Snail; green, Pika; brown, Urashima.

MALDI-TOF/MS fingerprinting of whole microbial cells was highly reproducible. A peak at m/z 9678 in the MALDI-TOF/MS spectra was detected in all strains despite their geographical origin (Figure 6). Some peaks were detected in some strains with relatively low intensities. Cluster analysis based on the presence or absence of peaks identified two clusters that would correspond to the geographical regions of isolation (Figure 7). Two *Persephonella* trees, one generated from the whole-cell MALDI-TOF/MS data and a ML tree from concatenated MLSA sequences, show similar topologies (Figure 7).

## Discussion

Here we investigated the microdiversity and phenotypic heterogeneity of extremely thermophilic chemolithoautotrophic bacteria in deep-sea hydrothermal vents. Genetic and phenotypic differences corresponding to the geographic origins were discovered by the combined use of MLSA and whole-cell MALDI-TOF/MS fingerprinting. The biogeography of hydrothermal vent-associated microbial community has been well studied (Takai et al., 2004; Nakagawa et al., 2005a,b; Kato et al., 2010). Members of the genus *Persephonella* have been found in global hydrothermal vent fields, however, their genetic and phenotypic heterogeneities were poorly understood.

### Genetic difference between OT and SMT populations

We identified 35 STs among 36 *Persephonella* strains by MLSA based on 6 protein-coding genes, indicating high genetic diversity of *Persephonella* population. The same ST is rarely shared among *Persephonella* strains, however, all SMT strains have the same alleles with other SMT strains but not with OT strains in one or more MLSA loci, suggesting that OT and SMT populations are significantly different. Likewise, two OT strains, i.e., OT-1 and -4, have the same allele (no. 7) at *napA* (Table 4), although the number of OT strains obtained in this study is small.

The split decomposition tree showed the evidence of recombination (Figure 3), which might contribute to increased STs. Previous studies showed that recombination generated the large number of unique combinations of alleles in some archaea and bacteria (Suerbaum et al., 2001; Whitaker et al., 2005; Doroghazi and Buckley, 2010).

### Biogeography of *Persephonella*

The phylogenetic analysis based on concatenated gene sequences separated the strains into two clusters according to their geographic origins (Figure 4). The *F*_ST_ value supported significant biogeographical isolation between SMT and OT populations. These results indicate that ubiquitous occurrence of *Persephonella* in deep-sea vents has not resulted from widespread contemporary dispersal but is an ancient historical legacy.

The microbial distribution seems to be not only influenced by local environmental conditions (Martiny et al., 2006). In this study, we observed clear correlation between the genetic distance and the geographic distance of isolates (Figure 5) as described in thermophilic archaea (Whitaker et al., 2003; Flores et al., 2012). On the contrary, genetic distance has no significant correlation with the difference in vent fluid pH and temperature. We cannot rule out the possibility that other factors not determined in this study, including grazing pressure and virus activity, may be correlated with the genetic difference of *Persephonella.* Recently, H_2_ concentration in vent fluids was shown to have an impact on the formation of microbial community structures in deep-sea vents (Takai and Nakamura, 2011).

### Correlation between genotypic and phenotypic heterogeneity

Some peaks in the MALDI-TOF/MS spectra were shared among some *Persephonella* strains. Major peaks of whole-cell MALDI-TOF/MS analysis are considered to reflect ribosomal proteins (Fenselau and Plamen, 2001; Ryzhov and Fenselau, 2001) and thus are independent of growth conditions (Bernardo et al., 2002). There were also some minor peaks that were specific to SMT or OT strains. Likewise the concatenated nucleotide alignment of MLSA loci, MALDI-TOF/MS data clustered the strains into two distinct groups corresponding to the geographic regions (Figure 7), suggesting that protein expression of *Persephonella* is tuned to function optimally in their original habitats. The genotypic and phenotypic correlation found among *Persephonella* isolates indicates the occurrence of allopatric speciation.

## Conclusion

By using both comparative genetic and phenotypic population characterizations, this study for the first time indicated the *Persephonella* populations were geographically distinct. Since the *Persephonella* members are extremely thermophilic chemoautotrophs endemic to deep-sea vents, considerable dispersal barriers for the migration to spatially distinct niches should exist. Focal points raised by this study for future research include the effects of cold, oxic deep-sea conditions on the viability of deep-sea vent (hyper) thermophiles during the dispersal, the biogeographical comparison with other ubiquitous thermophiles with different metabolic traits (e.g., heterotrophic fermenters and methanogens), and the comparison with moderately thermophiles or mesophiles with similar energy/carbon metabolisms (e.g., *Epsilonproteobacteria)* in deep-sea vents.

### Conflict of interest statement

The authors declare that the research was conducted in the absence of any commercial or financial relationships that could be construed as a potential conflict of interest.
